# The Stromal Processing Peptidase of Chloroplasts is Essential in *Arabidopsis*, with Knockout Mutations Causing Embryo Arrest after the 16-Cell Stage

**DOI:** 10.1371/journal.pone.0023039

**Published:** 2011-08-16

**Authors:** Raphael Trösch, Paul Jarvis

**Affiliations:** Department of Biology, University of Leicester, Leicester, United Kingdom; University of Oxford, United Kingdom

## Abstract

Stromal processing peptidase (SPP) is a metalloendopeptidase located in the stroma of chloroplasts, and it is responsible for the cleavage of transit peptides from preproteins upon their import into the organelle. Two independent mutant *Arabidopsis* lines with T-DNA insertions in the *SPP* gene were analysed (*spp-1* and *spp-2*). For both lines, no homozygous mutant plants could be detected, and the segregating progeny of *spp* heterozygotes contained heterozygous and wild-type plants in a ratio of 2∶1. The siliques of heterozygous *spp-1* and *spp-2* plants contained many aborted seeds, at a frequency of ∼25%, suggesting embryo lethality. By contrast, transmission of the *spp* mutations through the male and female gametes was found to be normal, and so gametophytic effects could be ruled out. To further elucidate the timing of the developmental arrest, mutant and wild-type seeds were cleared and analysed by Nomarski microscopy. A significant proportion (∼25%) of the seeds in mutant siliques exhibited delayed embryogenesis compared to those in wild type. Moreover, the mutant embryos never progressed normally beyond the 16-cell stage, with cell divisions not completing properly thereafter. Heterozygous *spp* mutant plants were phenotypically indistinguishable from the wild type, indicating that the *spp* knockout mutations are completely recessive and suggesting that one copy of the *SPP* gene is able to produce sufficient SPP protein for normal development under standard growth conditions.

## Introduction

The chloroplast is a unique plant cell compartment which harbours many essential processes such as photosynthesis, starch metabolism, and the biosynthesis of lipids and secondary metabolites [1], [2]. Like all plastids, chloroplasts are derived from an ancient free-living cyanobacterial ancestor that was incorporated into early eukaryotic cells through endosymbiosis [3]. As a result of this evolutionary origin, modern chloroplasts contain DNA and are able to synthesize roughly one hundred of their own proteins [4]. Nonetheless, the bulk of the ∼3000 different proteins in chloroplasts are encoded in the nuclear genome and must be imported post-translationally from the cytosol [5], [6].

Soon after the emergence of the signal hypothesis to account for the translocation of ER proteins, it was suggested that nucleus-encoded chloroplast proteins are similarly synthesized with a targeting tag that directs them to the organelle [7], [8]. This tag is an N-terminal extension of the protein called a transit peptide, and it is cleaved off after organellar import, producing a smaller, mature form of the chloroplast protein [9]. Chloroplast transit peptides vary greatly in length and amino acid sequence, and while secondary structural features have been reported in some cases the general significance of such observations remains uncertain [10], [11]. Thus, it is not fully understood how different preproteins are all targeted quite specifically to the same organelle. Transit peptides do contain slightly more hydroxylated residues and fewer acidic residues than average, giving them a net positive charge, and it has been suggested that a lack of a secondary structure might be necessary for their targeting properties [12].

The transit peptide is recognized by receptor components at the chloroplast surface, and subsequently the preprotein is guided through pores in the outer and inner envelope membranes. The multiprotein assemblies responsible for these recognition and translocation events are the TOC and TIC complexes (translocon at the outer/inner envelope membrane of chloroplasts) [13], [14], [15], [16], [17]. Upon reaching the stromal side of the envelope, the transit peptide is removed by the stromal processing peptidase (SPP), a metalloendopeptidase of the M16 family (members of which include subunit β of the mitochondrial processing peptidase, MPP, and *Escherichia coli* pitrilysin) which has a high specificity for chloroplast transit peptides [18], [19], [20]. The SPP enzyme recognizes a stretch of basic residues with weak sequence or physicochemical conservation at the C-terminus of the transit peptide [21], [22], [23]. Following recognition, it cleaves the transit peptide from the mature sequence using the catalytic activity of its zinc-binding domain, and subsequently proteolyses the C-terminal binding site of the transit peptide which facilitates release of the peptide fragments so that they may be degraded by the presequence protease, PreP [22], [24], [25]. Homologues of SPP exist in red and green algae as well as in the malaria parasite, *Plasmodium falciparum*, suggesting that the protein's function is well conserved amongst plastid-containing organisms [20]. An ancestral activity was probably inherited with the original endosymbiont, as SPP-related sequences even exist in cyanobacteria.

Antisense mediated down-regulation of *SPP* gene expression in *Arabidopsis* or tobacco plants resulted in chlorotic, albino or even a seedling-lethal phenotypes, indicating that the SPP enzyme plays an important role in chloroplast biogenesis [26], [27]. Indeed, the antisense lines displayed reduced numbers of chloroplasts per cell, and those organelles that were present were structurally abnormal. Both *in vitro* import experiments (using isolated chloroplasts and radiolabelled preproteins) and an *in vivo* targeting assay (involving expression of a transit peptide fusion to green fluorescent protein) revealed defects in chloroplast protein import in the antisense lines [26], [27]. Such defects may reflect the fact that most components of the TOC-TIC import machinery are themselves synthesized as preproteins (with transit peptides that presumably must be removed before those components can begin to operate), or indicate that transit peptide cleavage is a fully-integrated step of the translocation mechanism. More recently, a hypomorphic *spp* allele was identified in a forward-genetic screen of ethyl methanesulfonate-mutagenized rice plants [28]. The relevant mutant lacks a conserved glutamate residue in a C-terminal M16 domain of SPP, and it exhibits chlorosis associated with small, underdeveloped chloroplasts as well as defective root development.

The aforementioned *in vivo* studies all analysed the consequences of reduced levels of SPP activity for plant development. To determine the consequences of complete loss of SPP protein, we identified and characterized T-DNA knockout mutants in the *Arabidopsis* background.

## Results and Discussion

**Figure 1:**
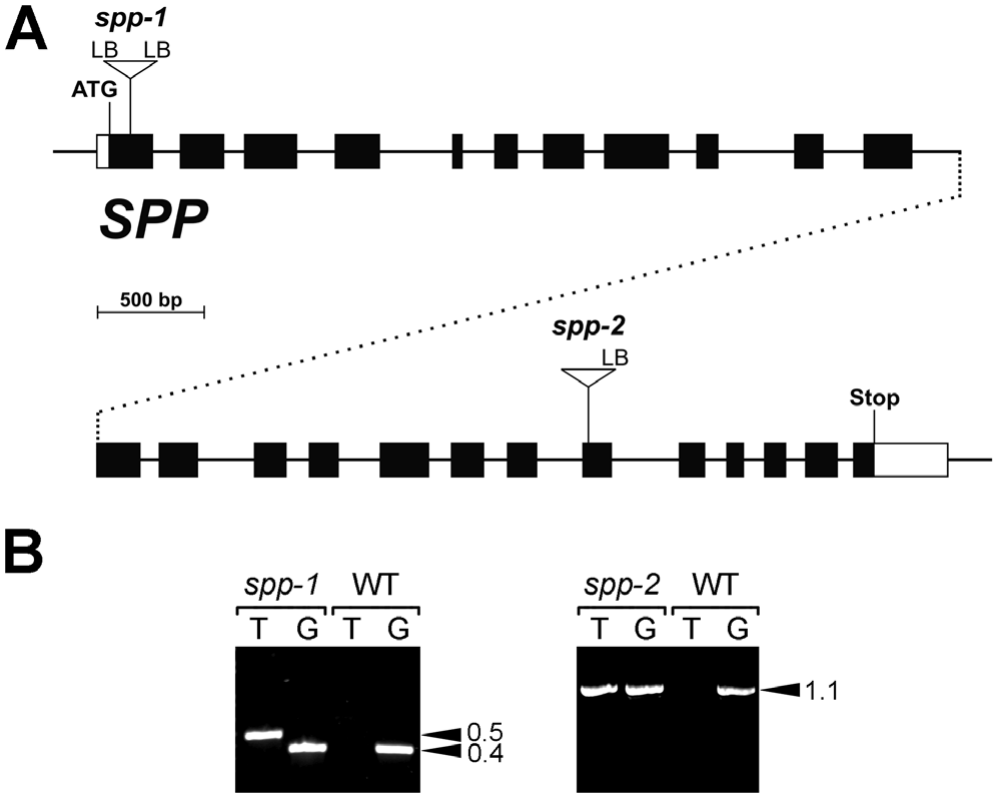
Molecular characterization of the *SPP* T-DNA insertion mutants. (A) Schematic diagram of the *SPP* gene showing the location of each T-DNA insertion. Protein-coding exons are represented by black boxes and untranslated regions by white boxes; introns are represented by thin lines between the boxes. The gene model shown is based on the RIKEN full-length cDNA clone, RAFL07-08-B09; it is separated over two lines (with the division at an arbitrary point near the middle, after intron 11; dotted lines) to aid presentation. Positions of T-DNA insertions are indicated precisely, but the insertion sizes are not to scale. ATG, translation initiation codons; Stop, translation termination codon; p(A), polyadenylation site. (B) Mutant genotype analysis by PCR. Genomic DNA samples extracted from wild-type and mutant plants (*spp-1* and *spp-2*) were analysed by PCR. Appropriate T-DNA- and *SPP*-gene-specific primers were employed in two different combinations: the first (T) comprised one T-DNA primer (LB) and one gene-specific primer (forward for *spp-1*; reverse for *spp-2*); the second (G) comprised two gene-specific primers flanking the T-DNA insertion site. The results shown for *spp-1* and *spp-2* are representative of those obtained for all antibiotic-resistant plants tested; amplification using both T and G indicated the presence of both mutant and wild-type alleles, respectively, and demonstrated that the plants were heterozygous. Amplicon sizes are indicated at right (in kb).

To further assess the importance of SPP during plant development, two different *Arabidopsis* lines with T-DNA insertions in the *SPP* gene (At5g42390) were obtained from the Nottingham Arabidopsis Stock Centre (NASC). These mutants were called *spp-1* (SALK_087683) and *spp-2* (SAIL_242_H11). Firstly, both T-DNA insertion sites were confirmed by genomic PCR, and by sequencing the T-DNA/gene junctions at one or both sides, as indicated (Figure 1A). In *spp-1*, the T-DNA disrupts the first exon of the *SPP* gene, whereas in *spp-2* the insertion lies in the nineteenth exon (Figure 1A).

Next, we attempted to identify homozygous mutant lines in each case, by genotyping 30 antibiotic-resistant plants for each line in PCR reactions using gene- and T-DNA-specific primers. However, when we did this, we found that all of the 60 tested plants (30 for each allele) were hemizygous for the relevant T-DNA insertion (Figure 1B), suggesting that the homozygous genotypes are not viable. Consistent with this notion, when segregation of the antibiotic-resistance marker associated with each T-DNA insertion was analysed (by plating seeds from heterozygous plants on medium containing either kanamycin [*spp-1*] or phosphinothricin [*spp-2*]), significant deviations from standard Mendelian inheritance were observed: only two antibiotic-resistant plants were observed for every one antibiotic-sensitive plant, instead of the more normal 3∶1 segregation ratio (Table 1).

**Table 1:** Segregation of the T-DNA-borne antibiotic resistance markers in the *spp* mutant lines.

Parental genotype	Plant number	Antibiotic	Resistant (R)	Sensitive (S)	R∶S ratio	*P*-value^a^
+/*spp-1*	1	Kanamycin	87	37	2.35	0.41
	2		82	44	1.86	0.71
	3		99	42	2.36	0.37
	4		69	46	1.50	0.13
	*sum*		337	169	1.99	0.97
+/*spp-2*	1	Phosphinothricin	74	38	1.95	0.89
	2		105	43	2.44	0.27
	3		78	43	1.81	0.61
	4		100	45	2.22	0.56
	5		83	40	2.08	0.85
	*sum*		440	209	2.11	0.54

a Goodness of fit of the observed ratios to 2∶1 was assessed by χ^2^ analysis. *P*-values are the probabilities that the observed ratios differ from 2∶1 due to random chance only.

**Figure 2:**
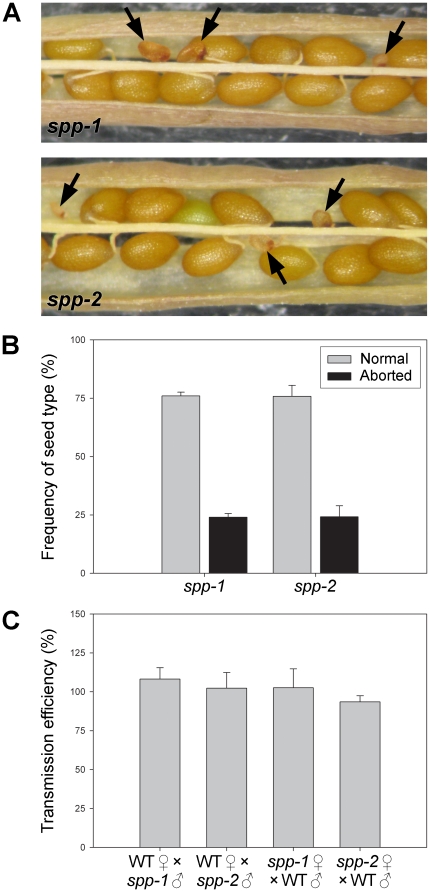
Embryo lethality of the *spp-1* and *spp-2* mutations. (A) Appearance of aborted seeds within mature siliques of heterozygous *spp-1* and *spp-2* plants. Aborted seeds indicative of embryo lethality (see black arrows) are smaller in size than normal seeds, and have a darker, shrivelled appearance. (B) Frequencies of normal and aborted seeds within ripe siliques of *spp-1* and *spp-2* heterozygotes. The data shown are means (±SD) derived from analyses of six different siliques per genotype, each one from a different plant. Values shown refer to fertilized seeds only. (C) Reciprocal crossing analysis. Transmission of the *spp-1* and *spp-2* mutations through the male and female gametes was assessed by crossing heterozygotes of both mutants to wild type, in both directions, multiple times (28–50 crosses using 3–4 different plants were conducted per direction, per allele). Inheritance of the *spp* mutations in the F_1_ progeny was assessed by determining antibiotic resistance of the F_1_ plants (kanamycin for *spp-1*; phosphinothricin for *spp-2*). Transmission efficiencies were calculated for each plant as described previously [31], and then these values were used to derive the means shown (±SD).

To investigate the possibility of embryo lethality, we carefully examined the seeds within ripe siliques of heterozygous *spp-1* and *spp-2* plants. Significant numbers of small, aborted seeds were observed in both mutant genotypes (Figure 2A), but not within wild-type siliques (data not shown). Amongst the fertilized seeds, abortions occurred with a frequency of almost exactly 25% (Figure 2B), strongly supporting the notion that the homozygous mutant genotypes were responsible for developmental arrest. Small numbers (∼3–5%) of what appeared to be failed ovules were also apparent in the *spp* siliques (data not shown), suggesting that there might be an additional effect of the mutations on gametophytic transmission [29], [30]. To assess this possibility, we conducted reciprocal crossing experiments, between both *spp* alleles and wild-type plants, analysing transmission of the mutations to the resulting F_1_ progenies by plating on selective media. However, the results revealed essentially normal transmission of both mutations through both male and female gametes (Figure 2C) [31]. This indicated that the *spp*-mediated block in development is exclusively post-fertilization, occurring during embryogenesis, and that the presumed failed ovules observed in ripe siliques (mentioned earlier) were perhaps very early seed abortions and/or the consequences of environmental stresses.

It is well documented that the disruption of chloroplast functions can lead to a block in embryogenesis [32]. In fact, it has been estimated that a disproportionately large number (∼25–30%) of non-redundant, embryo-lethal mutations in *Arabidopsis* affect chloroplast proteins [33], [34]. Prominent amongst the chloroplast functions that lead to embryo arrest, when disrupted, are: plastid gene expression (including RNA and protein synthesis); non-photosynthetic metabolism (including amino acid, vitamin and nucleotide biosynthesis); and, protein modification, transport and degradation [32], [34]. Of the previous reports linking chloroplast function to embryogenesis, those pertaining to two core components of the chloroplast protein import machinery, atToc75-III and atTic110, are most relevant [35], [36], [37], [38]. The aborted seeds observed in the *spp* mutant siliques (Figure 2A) appeared to be somewhat larger than those in *toc75-III* [35], and smaller in size than those in *tic110* siliques [37], suggesting that the *spp* mutations may affect embryo development at a stage intermediate between *toc75-III* and *tic110*.

**Figure 3:**
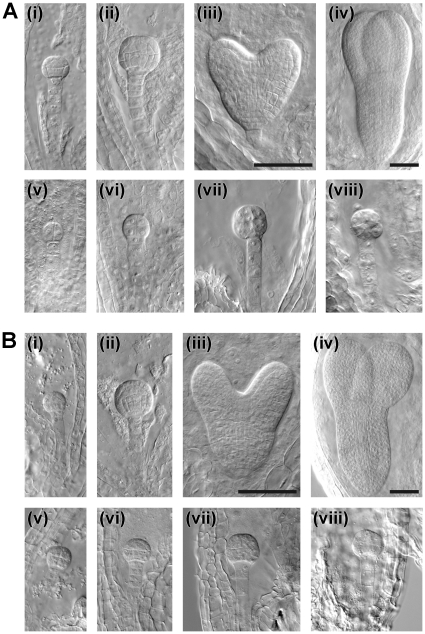
Analysis of embryo development in the *spp* mutants using Nomarski optics. Equivalent developmental series for normal (i–iv) and mutant (v–viii) embryos within immature heterozygous siliques of *spp-1* (A) and *spp-2* (B). Normal embryos: i, 16-cell stage; ii, early globular stage; iii, heart stage; iv, torpedo stage. Corresponding mutant embryos from the same siliques: v, 2- to 8-cell stages; vi, 8- to 16-cell stages; vii and viii, arrested or abnormal 16-cell stages. Embryo developmental stage names refer to the cell number or morphology of the embryo proper. Images i–iii and v–viii are all at the same magnification (40× objective); images iv are at lower magnification (20× objective). Bars = 50 µm.

To precisely determine the stage of embryogenesis during which the *spp* mutations arrest growth, we conducted a detailed examination of developing embryos in both mutants as well as in the wild type, using Nomarski optics microscopy (Table 2). Figure 3 shows equivalent developmental series for normal (i–iv) and mutant (v–viii) embryos within immature siliques of self-pollinated *spp-1* (Figure 3A) and *spp-2* (Figure 3B) heterozygotes. Typically, when normal embryos were at the 16-cell stage (Figure 3, i panels), mutant embryos were delayed at the 2- to 8-cell stages (Figure 3, v panels). As the normal embryos progressed to the globular and heart stages (Figure 3, ii and iii panels), the mutants were retarded at the 8- to 16-cell stages (Figure 3, vi and vii panels). In fact, the mutant embryos were never seen to develop normally beyond the 16-cell stage, even when the other embryos had reached the torpedo stage (Figure 3, iv and viii panels). Cell boundaries in the most mature mutant embryos were frequently indistinct, and such embryos often had an irregular or swollen appearance. As the normal seeds reached the cotyledon stage, significant numbers of aborted seeds (with degenerated structures containing no discernable embryo) became apparent within the mutant siliques (Table 2). In contrast with the situation in *spp* siliques, where two distinct classes of embryos could be observed (normal and mutant; the latter corresponding to ∼25% of the total), embryos within individual wild-type siliques rarely spanned more than three consecutive developmental stages (Table 2).

**Table 2:** Distribution of embryo phenotypes in single siliques of wild type and *spp* mutant heterozygotes.^a^

Genotype	Silique number^b^	1-cell	2-cell	8-cell	16-cell	Aborted seed^c^	EG	LG	EH	Heart	Torpedo	Cotyledon	Total scored	Fraction delayed or abnormal
**WT**	2	-	-	9	22	-	5	-	-	-	-	-	36	-
	4	-	-	-	4	-	11	23	6	-	-	-	44	-
	6	-	-	-	-	-	4	33	10	-	-	-	47	-
	8	-	-	-	-	-	-	20	18	9	-	-	47	-
	10	-	-	-	-	-	-	-	17	33	-	-	50	-
	12	-	-	-	-	-	-	-	-	37	18	-	55	-
	14	-	-	-	-	-	-	-	-	1	19	33	53	-
	16	-	-	-	-	-	-	-	-	-	-	53	53	-
***spp-1***	2	2	15	22	5	-	-	-	-	-	-	-	44	-
	4	-	2	12	25	-	2	-	-	-	-	-	41	-
	6	1	3	7	17	-	17	-	-	-	-	-	45	-
	8	-	-	3	12	-	3	15	15	-	-	-	48	0.31
	10	-	2	2	8	-	-	5	4	21	-	-	42	0.29
	12	-	-	-	8	8	-	-	-	5	22	2	45	0.36
	14	-	-	-	6	6	-	-	-	-	3	26	41	0.29
***spp-2***	2	-	2	12	6	-	4	-	-	-	-	-	24	-
	4	-	-	12	11	-	7	1	1	-	-	-	32	-
	6	-	-	4	6	-	12	5	3	-	-	-	30	-
	8	-	-	8	2	-	2	10	13	-	-	-	35	-
	10	-	-	2	5	1	9	5	6	10	-	-	38	0.21
	12	-	-	2	8	2	-	-	-	15	21	-	48	0.25
	14	-	-	-	2	7	-	-	-	2	30	-	41	0.22
	16	-	-	-	1	9	-	-	-	-	6	26	42	0.24

a Embryo developmental stage names refer to the cell number or morphology of the embryo proper. Abbreviations are defined as follows: EG, early globular; LG, later globular; EH, early heart.

b Siliques were numbered consecutively from the top of the inflorescence, such that the oldest siliques have the highest numbers.

c Aborted seed were shrivelled with no visible embryo or endosperm development.

Thus, the *spp* mutations indeed arrest embryogenesis at a stage (the 16-cell stage) intermediate between those during which *toc75-III* (two-cell stage) and *tic110* (globular stage) block growth [35], [36], [37], [38]. These differences in phenotype severity may partly reflect the differing expression patterns of the genes during embryogenesis: publicly-available microarray data show that *atTOC75-III* expression peaks at a much earlier stage in embryogenesis than that of either *atTIC110* or *SPP* (Figure S1) [39], [40], [41]. The phenotypic differences may also be linked to the roles of the proteins, as has been discussed [34]. Toc75 is the channel component of the outer membrane through which the vast majority of chloroplast proteins gain entry, including many envelope proteins, while Tic110 is thought to play roles in channel formation and/or the coordination of chaperones at the inner membrane, and is likely to be utilized by a smaller subset of chloroplast-destined proteins [16], [17]. The proteins that are dependent upon SPP for proper targeting may be intermediate in number and/or importance. An alternative explanation is that the differing phenotypes are a reflection of differing stabilities of the proteins, and the extent to which they can persist at functional levels during embryogenesis following the segregative loss of a functional gene. Irrespective of the basis for these differences in severity, the data leave no doubt that all three proteins play crucial roles in protein transport, and that efficient protein transport into plastids is essential during embryogenesis.

**Figure 4:**
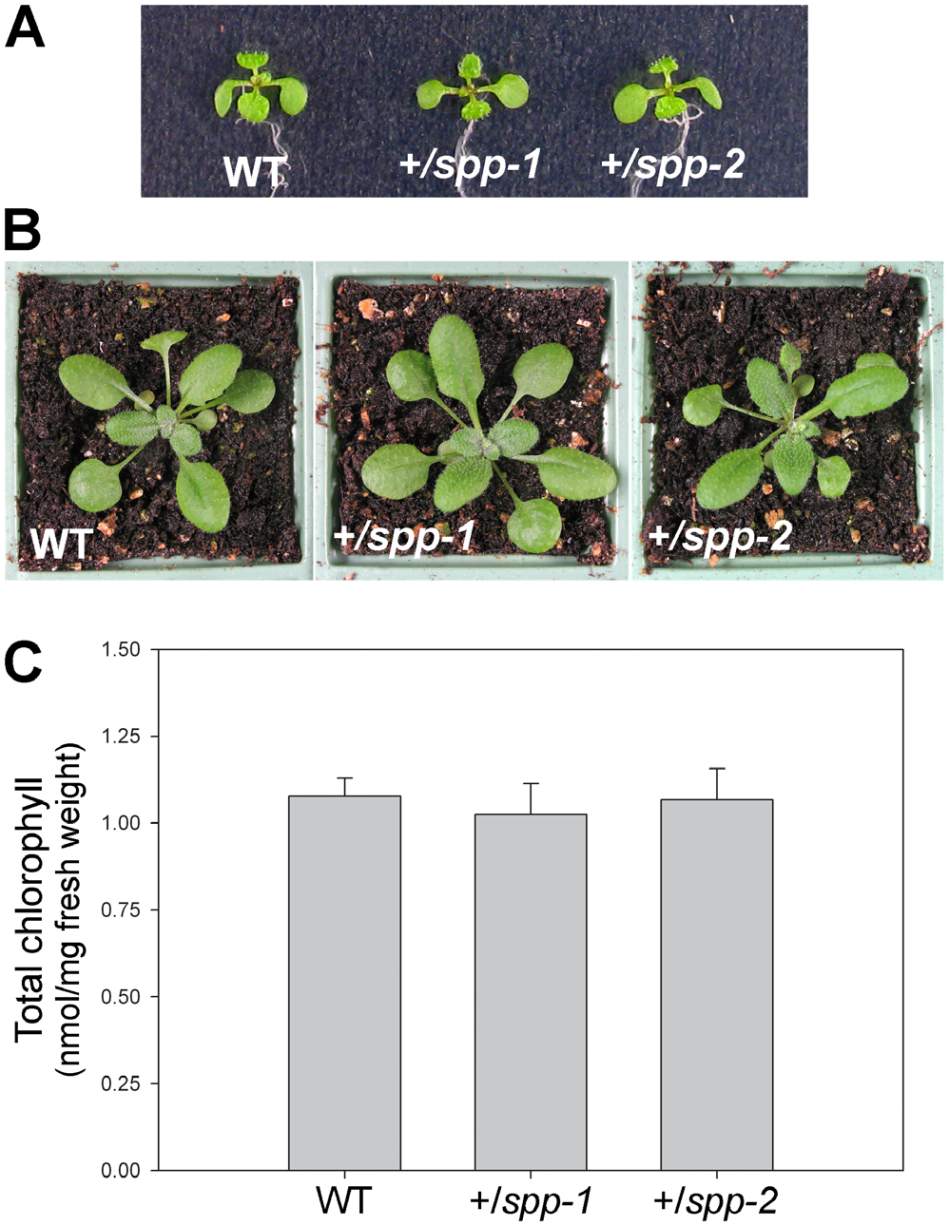
Phenotypic analysis of the *spp* heterozygotes. (A, B) Plants of the indicated genotypes were grown *in vitro* on selective medium (non-selective in the case of wild type) for 7 days, and then photographed (A). Additional similar plants were transferred to soil on day 7, and then allowed to grow further until they were 21 days old prior to photography (B). Representative plants are shown in each case. (C) Chlorophyll concentrations in 21-day-old plants grown as described above were determined using a SPAD-502 meter. Values shown are means (±SD) derived from six independent measurements per genotype, each one taken using a different plant. Units are nmol chlorophyll *a*+*b* per mg fresh weight of tissue.

While *toc75-III* mutations are completely recessive [35], heterozygous *tic110* plants are clearly chlorotic with quantifiable defects in chloroplast biogenesis [37]. In this respect, the *spp* mutations are more similar to *toc75-III*, as *spp* heterozygotes were visibly indistinguishable from wild type throughout development (Figure 4, A and B), and accumulated normal levels of chlorophyll pigment (Figure 4C). This indicates that a single copy of the *SPP* gene is able to produce sufficient quantities of the peptidase for normal growth under standard conditions. The phenotypic difference between *tic110* heterozygotes and *toc75-III* or *spp* heterozygotes cannot easily be explained in terms of mRNA or protein levels, as quantitative RT-PCR and immunoblot experiments did not reveal more severe deficiencies in +/*tic110* plants (Figure S2). As was discussed previously [42], the greater dosage dependency of the *tic110* mutation may reflect the absence of excess expression capacity for atTic110 in the wild type.

### Conclusion

While it was evident from earlier studies that SPP is an important chloroplast protein, the phenotype of homozygous knockout plants had not previously been reported. In this study, we employed *Arabidopsis* T-DNA insertion lines to demonstrate that SPP is indispensable *in vivo*. Homozygotes were absent from the progeny of plants carrying *spp* mutations, while a quarter of the seeds in the siliques of such plants were aborted, consistent with an embryo lethal phenotype. The mutant embryos exhibited delayed development, with cell divisions not terminating properly after the 16-cell stage. This work further emphasizes the importance of plastid functions during seed development, and extends the list of protein transport components known to be essential for plant viability.

## Materials and Methods

### Plant growth and chlorophyll analysis

All *Arabidopsis thaliana* plants were of the Columbia-0 ecotype. For *in vitro* growth, seeds were surface sterilized, sown on Murashige and Skoog agar medium containing 0.5% (w/v) sucrose in petri plates, cold treated at 4°C, and thereafter kept in a growth chamber, as described previously [43]. To select for the presence of T-DNA insertions, the following antibiotics were added to the medium: kanamycin monosulfate, 50 µg/ml (*spp-1*); phosphinothricin, 10 µg/ml (*spp-2*). All plants were grown under a long-day cycle (16 h light, 8 h dark).

Chlorophyll content was measured using a SPAD-502 meter following the manufacturer's instructions (Konica-Minolta, Osaka, Japan). Conversion from SPAD units to chlorophyll concentration values (nmol chlorophyll *a*+*b* per mg fresh weight) was done using a validated calibration curve [44].

### Identification of the *spp* mutants

The *spp-1* mutant was from the Salk Institute Genomic Analysis Laboratory (line number SALK_087683) [45], while the *spp-2* mutant was from Syngenta (line number SAIL_242_H11) [46]. Mutant genotypes were assessed by PCR (Figure 1B). Genomic DNA was extracted from *Arabidopsis* plants using a published protocol [47] and PCR was conducted using standard procedures. The primers used were as follows: *spp-1* forward, 5′-CTTCAAACCCTTTGCTACAAA-3′; *spp-1* reverse, 5′-GACGATGGATTAAACCTAACT-3′; *spp-1* T-DNA LB, 5′-GCGTGGACCGCTTGCTGCAACT-3′; *spp-2* forward, 5′-AAACTGTGTATAGGTCTGGTT- 3′; *spp-2* reverse, 5′-GGAGACGAGAGATGAGTATAGATAATGGGG-3′; *spp-2* T-DNA LB, 5′-TAGCATCTGAATTTCATAACCAATCTCGATACAC-3′. Amplification products were resolved by agarose gel electrophoresis and stained with SYBR Safe (Invitrogen). The location of each T-DNA insertion was determined precisely (Figure 1A) by sequencing PCR products spanning both junctions (except in the case of *spp-2*, where only one junction was identified).

### Seed and embryo analyses

The phenotypes of seeds in ripe siliques were determined by dissecting siliques held on double-sided sticky tape using a stereo microscope (Zeiss Stemi 2000). The analysis of cleared wild-type and *spp* mutant embryos using Nomarski optics was performed as described previously [35], [48], using a microscope equipped for differential interference contrast (Nikon Eclipse 80i).

## Supporting Information

Figure S1 **Expression patterns of essential chloroplast protein import apparatus genes during embryogenesis.** Publicly-available Affymetrix microarray data corresponding to defined tissues and developmental stages of Arabidopsis embryogenesis [39], [40] were accessed using an electronic fluorescent pictograph (eFP) browser online [41]. Data for *atTOC75-III* (At3g46740), *atTIC110* (At1g06950) and *SPP* (At5g42390) are shown. Values are means (±SE) derived from three or six independent measurements. SAM, shoot apical meristem.
(TIF)

Figure S2
**Analyses of mRNA and protein expression in the** ***toc75-III*** **,** ***tic110*** **and** ***spp*** **heterozygotes.** (A) Quantitative RT-PCR analysis of mRNA levels. Total-RNA samples were extracted from ∼10–30 whole seedlings of the indicated genotypes (and wild type) that had been grown *in vitro* for 14 days under standard conditions. RNA was isolated using an RNeasy Plant Mini Kit (Qiagen, Hilden, Germany), and was then treated with DNAse I (DNA-free; Ambion, Texas, USA). Quantitative RT-PCR was performed using an MJ Research Chromo4 Gradient Cycler (Bio-Rad, Hercules, CA, USA) and SYBR Green Jump Start Taq Ready Mix (Sigma, St. Louis, MO, USA) for high-throughput quantitative PCR. Relative quantification was determined according to published methods [49], [50]. Reactions were characterized by comparing threshold cycle (*C*_T_) values; *C*_T_ is a unit-less value defined as the fractional cycle number at which the sample fluorescence signal passes a fixed threshold. The relative amount of transcript was calculated by subtracting the control gene (*ACTIN 2*; At3g18780) *C*_T_-value from the gene-of-interest (*atTOC75-III*, *atTIC110*, *SPP*) *C*_T_-value (Δ*C*_T_). Then, Δ*C*_T_ values for the wild type were subtracted from those for the mutants to yield ΔΔ*C*_T_ values, and these were used to estimate expression levels as 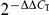. Data shown are means (±SE) derived from four independent amplifications performed on three biological replicates. The *ACTIN2* primers have been described previously [51]; the other primers used were: *atTOC75-III* sense 5′-TCG CAT CTC CAC TCA ATC-3′; *atTOC75-III* antisense, 5′-GTC TCT GTA TCT CGG TTA GG-3′; *atTIC110* sense, 5′-CTC CTC AGG TGC CTT ATC AGA AG-3′; *atTIC110* antisense, 5′-CGA GCA AGA GCA GCC GAG AAC-3′; *SPP* sense, 5′-AAG CTA GCC ATG ATT CTG CAA-3′; *SPP* antisense, 5′-CAT CAT GAG CAA CAG GAA GTT-3′. (B) Immunoblot analysis of protein levels. Total-protein was extracted from plant material samples equivalent to those employed in panel A, using previously-described procedures [51]. Three different protein amounts (80, 60 and 40 µg) of each genotype were analysed by immunoblotting, as described previously [51], using primary antibodies against atToc75-III, atTic110 and the plasma membrane H^+^-ATPase, PMA2 [51], [52], [53]; unfortunately, an SPP antibody was not available for this work. The secondary antibody was anti-rabbit IgG conjugated with horseradish peroxidase (Santa Cruz Biotechnology, Santa Cruz, CA, USA), and the detection reagent was ECL Plus (GE Healthcare, Chalfont St. Giles, UK). Chemiluminescence detection employed a Fujifilm LAS-4000 imager. Quantification of all images was performed using Aida software (Raytest, Straubenhardt, Germany). The atToc75-III and atTic110 data were normalized using equivalent PMA2 data. Values shown are means (±SE) derived from three independent measurements.(TIF)
