# Supplementary figures and images for: The Stromal Processing Peptidase of Chloroplasts is Essential in Arabidopsis, with Knockout Mutations Causing Embryo Arrest after the 16-Cell Stage
Source: PLoS One. 2011 Aug 16;6(8):e23039. doi: 10.1371/journal.pone.0023039 (PMC3156710; doi:10.1371/journal.pone.0023039)

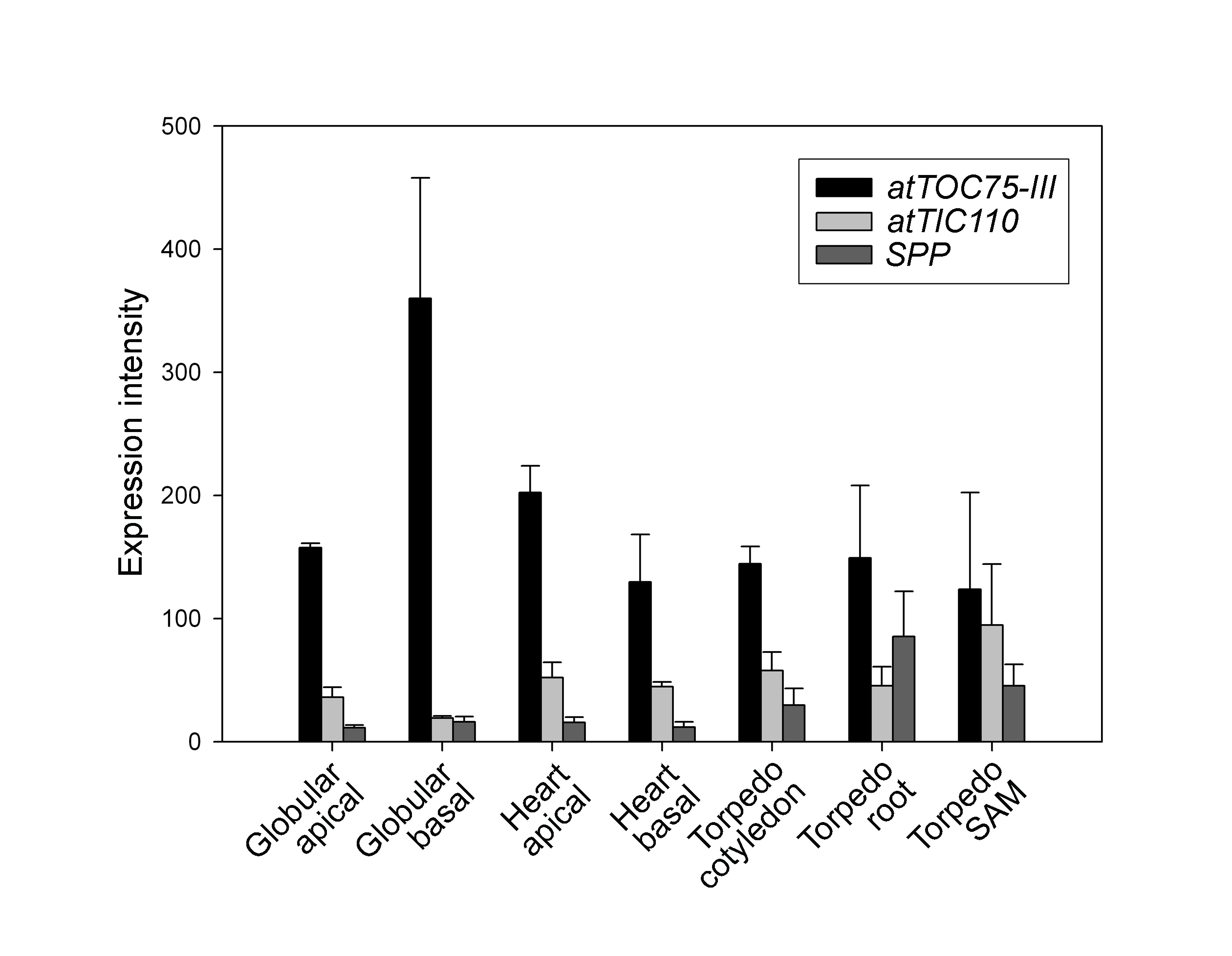

Supplement: Figure S1 — Expression patterns of essential chloroplast protein import apparatus genes during embryogenesis. Publicly-available Affymetrix microarray data corresponding to defined tissues and developmental stages of Arabidopsis embryogenesis [39], [40] were accessed using an electronic fluorescent pictograph (eFP) browser online [41]. Data for atTOC75-III (At3g46740), atTIC110 (At1g06950) and SPP (At5g42390) are shown. Values are means (±SE) derived from three or six independent measurements. SAM, shoot apical meristem. (TIF) [file pone.0023039.s001.tif]

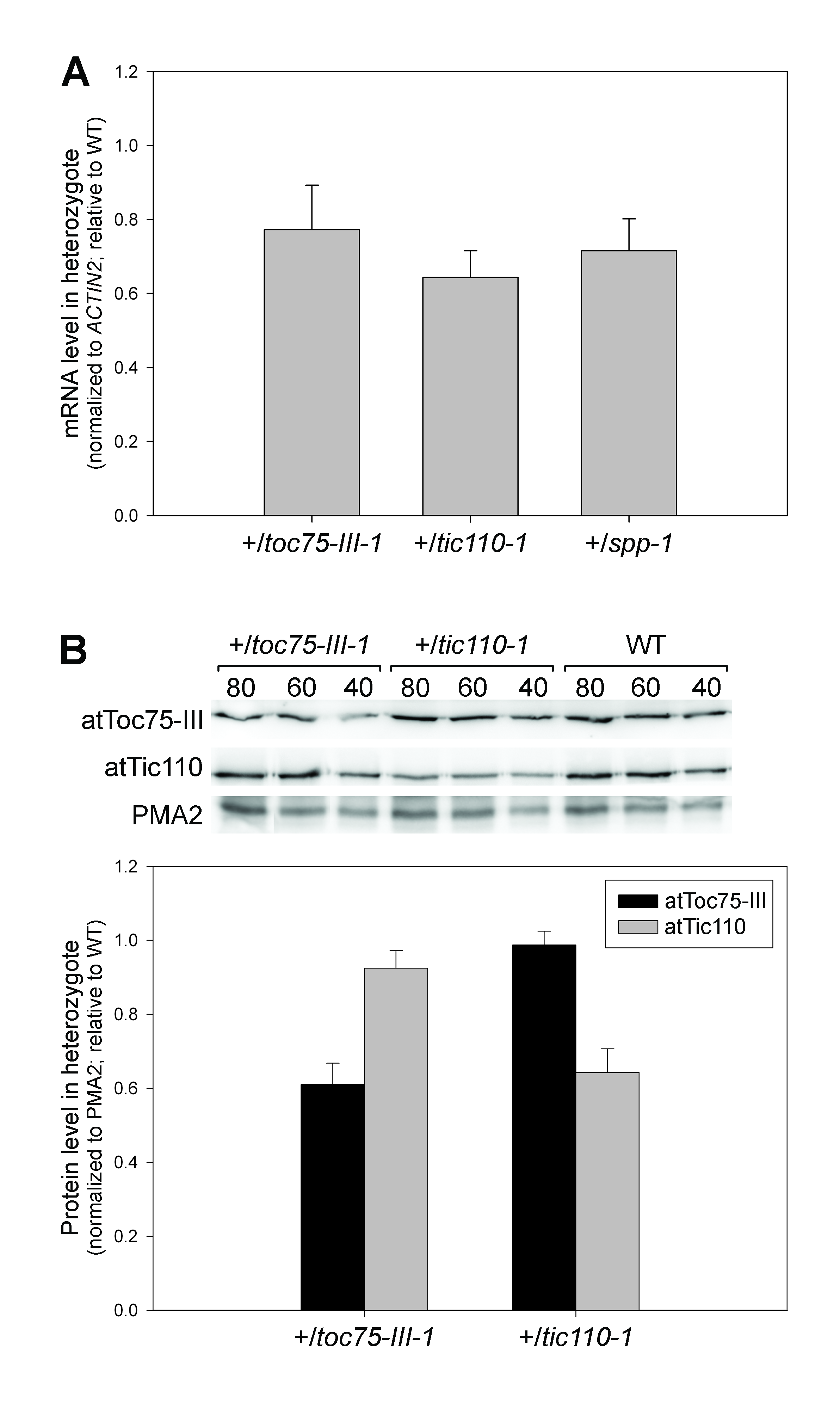

Supplement: Figure S2 — Analyses of mRNA and protein expression in the toc75-III , tic110 and spp heterozygotes. (A) Quantitative RT-PCR analysis of mRNA levels. Total-RNA samples were extracted from ∼10–30 whole seedlings of the indicated genotypes (and wild type) that had been grown in vitro for 14 days under standard conditions. RNA was isolated using an RNeasy Plant Mini Kit (Qiagen, Hilden, Germany), and was then treated with DNAse I (DNA-free; Ambion, Texas, USA). Quantitative RT-PCR was performed using an MJ Research Chromo4 Gradient Cycler (Bio-Rad, Hercules, CA, USA) and SYBR Green Jump Start Taq Ready Mix (Sigma, St. Louis, MO, USA) for high-throughput quantitative PCR. Relative quantification was determined according to published methods [49], [50]. Reactions were characterized by comparing threshold cycle (C T) values; C T is a unit-less value defined as the fractional cycle number at which the sample fluorescence signal passes a fixed threshold. The relative amount of transcript was calculated by subtracting the control gene (ACTIN 2; At3g18780) C T-value from the gene-of-interest (atTOC75-III, atTIC110, SPP) C T-value (ΔC T). Then, ΔC T values for the wild type were subtracted from those for the mutants to yield ΔΔC T values, and these were used to estimate expression levels as . Data shown are means (±SE) derived from four independent amplifications performed on three biological replicates. The ACTIN2 primers have been described previously [51]; the other primers used were: atTOC75-III sense 5′-TCG CAT CTC CAC TCA ATC-3′; atTOC75-III antisense, 5′-GTC TCT GTA TCT CGG TTA GG-3′; atTIC110 sense, 5′-CTC CTC AGG TGC CTT ATC AGA AG-3′; atTIC110 antisense, 5′-CGA GCA AGA GCA GCC GAG AAC-3′; SPP sense, 5′-AAG CTA GCC ATG ATT CTG CAA-3′; SPP antisense, 5′-CAT CAT GAG CAA CAG GAA GTT-3′. (B) Immunoblot analysis of protein levels. Total-protein was extracted from plant material samples equivalent to those employed in panel A, using previously-described procedures [51]. Three different [file pone.0023039.s002.tif]
